# Leakage Evaluation by Virtual Entropy Generation (VEG) Method

**DOI:** 10.3390/e20010014

**Published:** 2017-12-29

**Authors:** Zhichao Zhang, Corina Drapaca, Zhifeng Zhang, Shuaifang Zhang, Shimei Sun, Hui Liu

**Affiliations:** 1 School of Municipal and Environmental Engineering, Jilin Jianzhu University, Changchun 130118, China; 2 Department of Engineering Science and Mechanics, Pennsylvania State University, State College, PA 16802, USA; 3 Department of Mechanical and Nuclear Engineering, Pennsylvania State University, State College, PA 16802, USA

**Keywords:** virtual entropy generation (VEG) method, leakage evaluation, micro/nano-scale crack

## Abstract

Leakage through microscale or nanoscale cracks is usually hard to observe, difficult to control, and causes significant economic loss. In the present research, the leakage in a pipe was evaluated by the virtual entropy generation (VEG) method. In virtual entropy generation method, the “measured entropy generation” is forced to follow the “experimental second law of thermodynamics”. Taking the leakage as the source virtual entropy generation, a new pipe leakage evaluation criterion was analytically derived, which indicates that the mass leakage rate should be smaller than the pressure drop rate inside a pipe. A numerical study based on computational fluid dynamics showed the existence of an unrealistic virtual entropy generation at a high mass leakage rate. Finally, the new criterion was used in the evaluation of leakage available in the literature. These results could be useful for leakage control or industry criteria design in the future.

## 1. Introduction

Leakage is a gradual escape of a current from a transportation channel or a container. The current can be an electric charge, magnetic flux, information, heat or fluid flow, etc. The management and control of these leakages are important topics. Among various leakages, fluid leak in a pipe is one of the simplest forms, widely existing in biology, process industries, and engineering.

Leakage plays different roles, depending on the application. A minimized leakage rate is required in industries involving, for instance, water/oil/gas processing, or energy recovery/storage. In the chemical process industry, leakage of flammable, radioactive, or explosive fluids is dangerous and even disastrous. On another hand, leakage may play a positive role in biological transportation, like drug delivery, capillary exchange, or cellular membrane filtration.

Towards a better process control of the leakage, an important strategy is to design a reasonable leakage criterion. Usually, leakage through cracks at a microscale or nanoscale is invisible or difficult to identify, even though various technical efforts like non-destructive evaluation or contact mechanics analysis [1]. Therefore, most existing leakage criteria are commonly empirical and not clearly defined. In addition, traditional leakage criterion are hardly valid in multiscale leakage evaluation.

Recently, the virtual entropy generation (VEG) method has been used in the design of process evaluation criterion for the heat exchange process [2,3,4]. In this method, VEG was defined as [3] the entropy generation difference of a real-life measurable process and an ideal irreversible process. Under the constraint of a non-negative entropy generation measurement for a real-life thermodynamic process, corresponding evaluation criteria can be obtained. Compared to traditional evaluation methods [5], the VEG method always provides a physically “meaningful” solution [2,3,4].

From the thermodynamic perspective, leakage happens in a process such as heat transfer [6], fluid flow, electric power transmission [7], or information transfer [8]. Defining a leakage criterion equals defining the process criterion. Evaluation of a leakage process by the VEG method has not been conducted.

In the present research, a leakage evaluation criterion in a single pipe was derived using the VEG method analytically. The leakage induced virtual entropy generation was controlled by the flow process entropy generation. We used published experimental data and numerical simulations to show how to use this parameter in the evaluation and control of leakage. The present research method would provide suggestive criteria for leakage control and leakage detection systems.

## 2. The VEG Method

**Figure 1:**
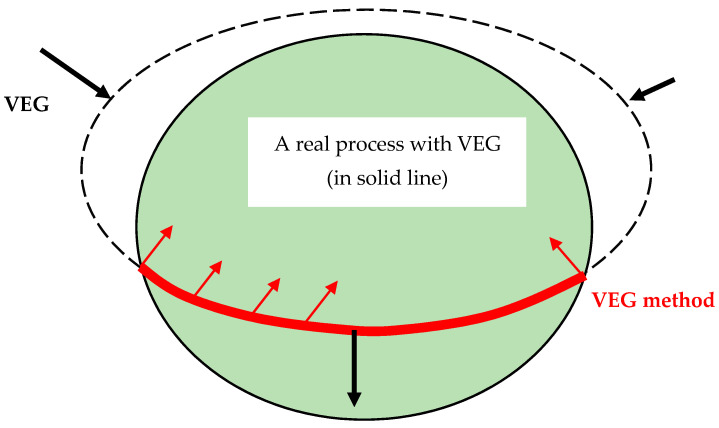
Schematic of the VEG method: the second law of thermodynamics for an ideal irreversible process (dashed line) and a real-life process (solid line) under the influence of VEG. The black arrows are sources of VEG: a non-thermodynamic process or defects in a mathematical model. The bold red line is the suggested VEG boundary experiments should follow. The red arrows are pointing towards a meaningful physical process.

Virtual entropy generation (VEG) is defined as a part of experimental entropy generation caused by factors that are outside the description of an ideal irreversible model. For example, such factors are any non-thermodynamic process factors, like measurement errors, or factors that cause a model failure like an undetectable leakage. As seen in Figure 1, the black arrows stand for sources of VEGs such as measurement errors or model defects. These VEGs deform the entropy generation measurement from the black dashed line (ideal irreversible process) to the black solid line (real-life measured process). The virtual entropy generation method introduces “positive entropy generation” as a criterion (the solid red line) in order to filter invalid experiment cases and prevent an over-deformed measured. VEG method forces the measurement results to obey the adaptation of the second law of thermodynamics to experiments proposed in the red arrows direction [3].

Based on the VEG criterion, invalid negative entropy generation is not allowed in an experiment. A mathematical representation of the VEG method applied to leakage is given by

(1) $${\left({\dot{S}}_{gen}\right)}_{m}={\dot{S}}_{gen}\left(process\right)+{\dot{S}}_{gen}\left(leakage\right)\geq 0$$

Here, ${\left({\dot{S}}_{gen}\right)}_{m}$ is the “measured” entropy generation of a real process. It is through indirect measurement—defined as a mathematical combination of direct measurement quantities such as pressure, temperature, and mass flow rate, see [2]. $S_{gen}\left(process\right)$ is the entropy generation of an ideal process assuming no leakage, and $S_{gen}\left(leakage\right)$ is the virtual entropy generation caused by leakage, which is the control target in the present research. Equation (1) shows that the leakage induced VEG should be bounded by the entropy generation of a corresponding ideal process.

## 3. Analytical Model

Fluid leakage in a pipe flow is one of the most common forms of leakage caused by cracks in the wall. Cracks on the scale of micron or sub-micron in a pipe are mainly caused by corrosion, operation outside design limit, or damage. The crack commonly exists in the form of multiple paths or porous structures [9]. Leaking through a crack is most often difficult to detect and hard to predict.

For a pipe flow without leakage, the entropy generation of an “ideal irreversible” process is [10]

(2) $${\left({\dot{S}}_{gen}\right)}_{m}={\dot{m}}_{1}s_{out}-{\dot{m}}_{1}s_{in}\geq 0$$

where ${\dot{m}}_{1}$ is the mass flow rate at the inlet of the pipe, $s_{out}$ is the entropy at the outlet, and $s_{in}$ is the entropy at the inlet.

In an experiment, the value of entropy generation for a leaky pipe flow is determined by the entropy difference between the outlet and inlet. For a general leaky pipe [11], by assuming a decreased mass flow rate at the outlet (i.e., ${\dot{m}}_{1}={\dot{m}}_{2}+\delta \dot{m}$, $\delta \dot{m}>0$), the indirect measurement value of entropy generation in this leaky pipe can be expressed as

(3) $${\left({\dot{S}}_{gen}\right)}_{m}={\dot{m}}_{2}s_{out}-{\dot{m}}_{1}s_{in}$$

Further, Equation (3) can be decomposed as

(4) $$\begin{array}{cc} {\left({\dot{S}}_{gen}\right)}_{m}={\dot{m}}_{2}s_{out}-({\dot{m}}_{2}+\delta \dot{m})s_{in}= & {\dot{m}}_{2}\left(s_{out}-s_{in}\right)-\delta \dot{m}(s_{in}-0)\\ & ⎯⎯⎯⎯⎯⎯⎯⎯⎯⎯\;\;\;\;⎯⎯⎯⎯⎯⎯⎯⎯⎯\\ & \mathrm{Irreversibility}\;\;\;\mathrm{VEG} \end{array}$$

In Equation (4), the first term on the right-hand side expresses the irreversibility of the flow through a real pipe, while the second term is the VEG caused by leaking.

An “ideal reversible” pipe transportation process has a positive entropy generation due to irreversibility. However, the condition of the second-law of thermodynamics may be too harsh in some cases. For example, it is not easy to isolate a 10-km-long pipeline transportation process in a real engineering case due to multiple leakages. Also, it is not possible to identify all leaking points in this real process. The “measured” entropy generation may return “negative” or invalid.

From the virtual entropy generation perspective, the invalid “measured entropy generation” is an indicator of unreliability in a real-life process. It points out a direction we need go against. For the case of a single pipe leakage, ‘invalid entropy generation’ is caused by undetected leakage. To prevent the over-leakage, the virtual entropy generation criterion, ${\left({\dot{S}}_{gen}\right)}_{m}\geq 0$, may help control and evaluate leakage.

Due to the mass leakage ($\delta \dot{m}$), the irreversibility measured for a real process may decrease, even leading to a negative value in Equation (3). The decrease in entropy generation will not happen in an isolated system or an ideal invertible process without leakage. However, for a leaky pipe with multiple outputs, it is not possible to take all the leaking conditions into consideration. The model we are proposing in this paper exists in many real-life conditions such as in a long-distance gas/oil transportation or a diffusion based capillary exchange, where multiple leaky sites could arise beyond normal control. To prevent a non-physical measurement, a VEG criterion should be determined to prevent a serious leaking situation.

According to the first law of thermodynamics, the enthalpy for a single-phase flow can be written as

(5) dh=Tds+vdP

where dh is the enthalpy change. According to the first law of thermodynamics, the adiabatic flow condition happens in a rapid flow condition when the flow is much faster than the leaking condition. Usually, the mass leakage flow process is a rapid process. It is much faster than the heat transfer process. Thus, we can use the adiabatic approximation, with d*h* = 0; *T* is the temperature assumed as constant for the adiabatic process; ds is the variance of entropy generation; v=1/ρ is the specific volume of the fluid of mass density ρ; and dP is the pressure change along the pipe. Thus, for an adiabatic process, Equation (5) reduces to

(6) $$ds=-\frac{1}{T\rho }dP$$

The leaking process is characterized by a mass flow rate $\delta \dot{m}$ and a pressure drop from $P_{in}$ to $P_{atm}=0$. Integrating Equation (6) further gives

(7) $${\left({\dot{S}}_{gen}\right)}_{m}={\dot{m}}_{2}\int_{P_{in}}^{P_{out}}\left(-\frac{1}{\rho T}\right)dP-\delta \dot{m}\int_{P_{in}}^{0}\left(-\frac{1}{\rho T}\right)dP\geq 0$$

Dividing the constant term (−1/ρT) on both sides of Equation (7), a relation can be obtained in the form of

(8) $$\delta \dot{m}\leq \left({\dot{m}}_{1}-\delta \dot{m}\right)\frac{\Delta P}{P_{in}}$$

Here, Δ*P* is the pressure drop across the inlet and outlet. The pressure at outlet [12] changes due to leakage ($\Delta P_{L}$) and friction ($\Delta P_{f}$), i.e., $\Delta P=\Delta P_{f}+\Delta P_{L}$. The friction induced pressure drop is described by Darcy–Weisbach equation: $\Delta P_{f}=f\frac{L}{D}\frac{\rho V^{2}}{2}$. Here, *f* is the friction factor, *L* is the length of the main channel, *D* is the diameter of a pipe, and *V* is the bulk velocity of the fluid. For an ideal compressible gas, ${\dot{S}}_{gen}=\dot{m}\int_{P_{in}}^{P_{out}}\frac{v}{T}dP\approx \dot{m}R\frac{\Delta P}{P_{out}}$, *R* is the gas constant. By dividing *R* on both sides of Equation (7), Equation (8) is still valid. Thus Equation (8) is valid for both incompressible and compressible fluids.

For cases with a small mass leakage rate, $\delta \dot{m}\ll {\dot{m}}_{1}$ the mass leakage rate is bounded by the operation condition of the ideal process in the non-dimensional form of

(9) $$\delta \dot{m}/{\dot{m}}_{1}\leq \Delta P/P_{in}$$

The mass leakage criterion defined here is a variable criterion: a high mass leakage rate is acceptable for a long-distance transportation with a high pressure drop rate. For a short distance, or transportation of a fluid with low viscosity through a smooth surface pipe, the VEG criterion in Equation (9) suggests a small mass leakage rate ($\delta \dot{m}/{\dot{m}}_{1}$). For long-distance transportation of a fluid with high viscosity through a rough surface pipe, a relatively high mass leakage rate is acceptable. This provides a criterion to control the cross-channel-wall mass leakage rate by the pressure drop along the pipe. The inlet pressure is commonly determined by a designed transportation requirement, or is constant.

The velocity of fluid after the leakage site decreases. Thus, the pressure drop across the leakage becomes lower. The measurement of pressure difference from the inlet to the outlet, $\Delta P=P_{in}-P_{out}$, becomes smaller. Consequently, the outlet entropy generation flowrate at the outlet in Equation (3) decreases fast at a high leakage rate.

Here, we define a critical mass leakage rate, ${\left(\delta \dot{m}/{\dot{m}}_{1}\right)}_{c}=\Delta P/P_{in}$. By inserting the critical leakage rate back in the expression of experimental entropy generation in Equation (4), we can obtain

(10) $$\begin{array}{cc} {\left({\dot{S}}_{gen}\right)}_{m} & ={\dot{m}}_{2}(s_{out}-s_{in})-\delta \dot{m}\left(s_{in}-0\right)\\ & ={\dot{m}}_{2}(s_{out}-s_{in})-{\dot{m}}_{1}\frac{\Delta p}{P_{in}}\left(s_{in}-0\right)\\ & ={\dot{m}}_{2}(s_{out}-s_{in})-{\dot{m}}_{1}\frac{\Delta P}{P_{in}}\frac{P_{in}}{\rho T}=-\frac{\delta \dot{m}\Delta P}{\rho T}<0 \end{array}$$

Thus, the entropy generation measured is already negative at the critical mass leakage rate. As we noticed that Equations (1)–(10) have no scale limit. However, for a leakage problem, cracks are commonly at microscale or nanoscale.

**Figure 2:**
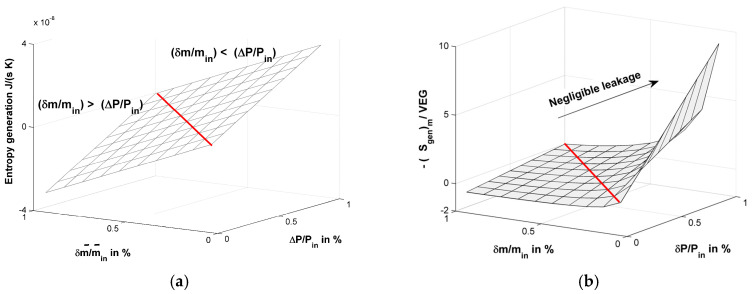
(**a**) Entropy generation measurement under leaking condition. Vertical axis unit is J/(s·K); (**b**) The relative entropy generation measurement number defined by $-{\left({\dot{S}}_{gen}\right)}_{m}/\delta \dot{m}s_{in}$. The *x* and *y* ordinate scales are in %.

In Figure 2a, the relation among ${\left({\dot{S}}_{gen}\right)}_{m}$, $\delta \dot{m}/{\dot{m}}_{1}$, and $\Delta p/p_{in}$ is plotted. As can be seen, when $\delta \dot{m}/{\dot{m}}_{1}>\Delta p/p_{in}$, the measured entropy generation is negative and increases linearly with $\delta \dot{m}/{\dot{m}}_{1}$ and $\Delta p/p_{in}$. Besides, we defined a non-dimensional form of entropy generation in Figure 2b by dividing the entropy generation measured by the undetectable virtual entropy generation $\delta \dot{m}s_{in}$. As can be seen on the right side of Equation (4), the non-dimensional form of entropy generation is the ratio of the first term and the second term. This ratio in the non-dimensional form provides a method to decrease the proportion of virtual entropy generation by increasing the pressure loss in the transportation at a constant inlet pressure condition.

## 4. Concept Validation by Computational Fluid Dynamics

**Figure 3:**
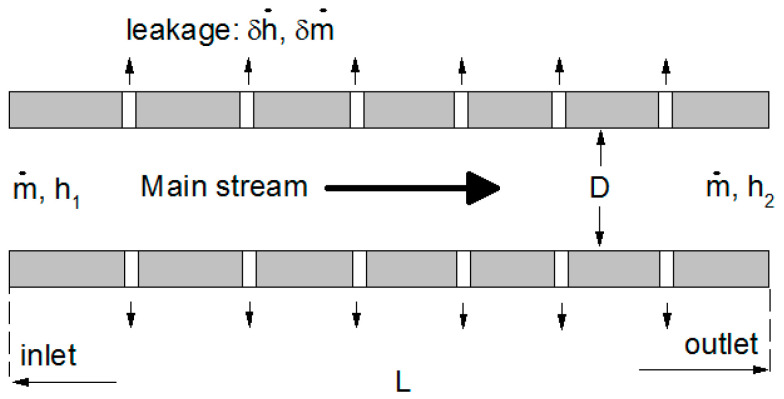
A leaky pipe model. This leaky pipe model is a multiple-output control volume system with a constant temperature and zero ambient pressure (as a reference) boundary. $P_{in}$ and $P_{out}$ are relative inlet and outlet pressures based on ambient pressure.

In this part, Computational Fluid Dynamic (CFD) simulations were conducted to show the existence of invalid entropy generation measurement in a leaky pipe. The numerical model was built based on the configuration in Figure 3. The main difference is that we focused on a single undetectable crack in the middle of the main channel. The main channel was set as 2 mm in height. The crack in the wall was modeled as a microchannel with three different widths of 50, 100, or 200 μm in height. The liquid viscosity is set as $\mu =10^{-3}\;\mathrm{Pa}\cdot s$ and the gas viscosity was set as $\mu =10^{-5}\;\mathrm{Pa}\cdot s$. Both liquid and gas are in laminar flow.

In the simulation, the pressure drop rate (*α*), which is the *x*-axis, was achieved by setting the pressure at the main channel inlet and outlet. The inlet boundary condition was defined as a stagnation inlet boundary (total pressure), and the outlet boundary condition was defined as a pressure outlet boundary (static pressure). The ambient pressure is assumed as 0-gauge. The pressure drop rate (*α*) steps from 1% to the value when the mass leakage rate (*β*) disobeys Equation (9). The inlet pressure of the main channel was set as a constant of 10 kPa for static pressure. The mass loss percentage (*β*), which is the *y*-axis, was collected from simulation results by solving the Navier–Stokes equation in the Standard CFD solver Ansys Fluent.

**Figure 4:**
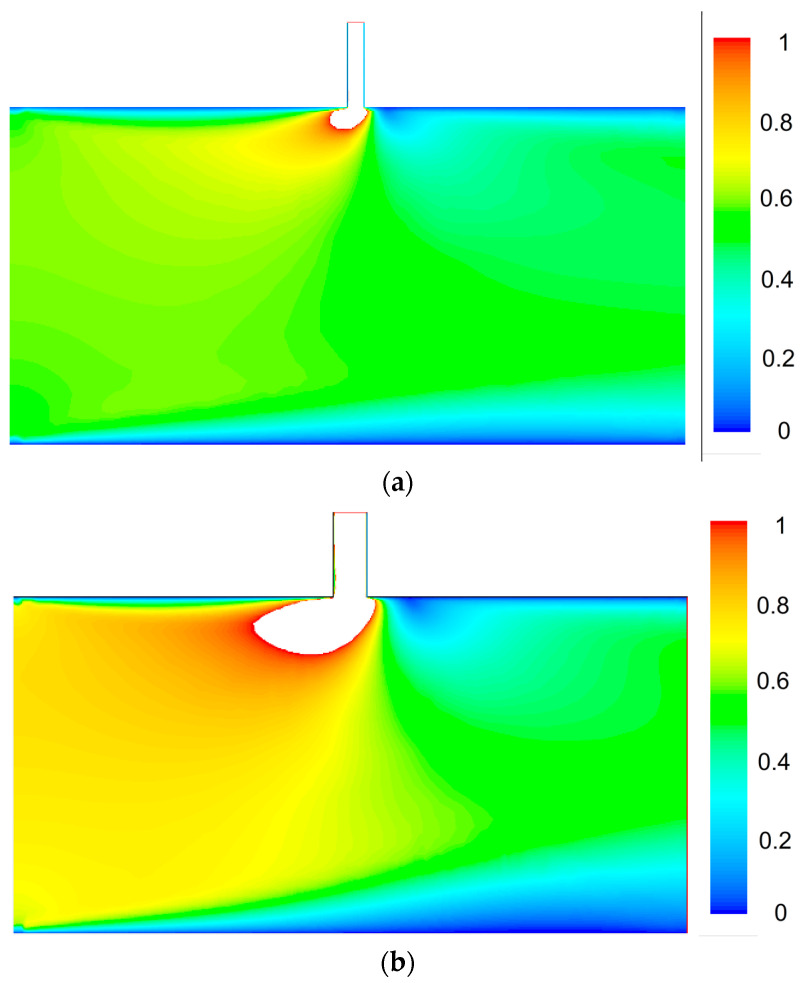
Velocity profile of a 2D liquid leakage channel with a pressure gradient of 20 kPa/m in the main channel for a, (**a**) 100 μm crack, and (**b**) 200 μm crack.

**Figure 5:**
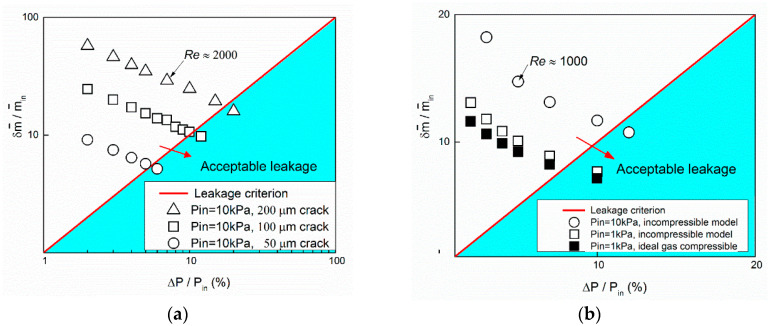
Pressure drop rate and mass leakage rate relation for the leakage of, (**a**) liquid, and (**b**) gas. Reynolds number is marked in each figure as reference.

By setting the pressure difference, the mass flow rate of two outlets (outlet 1 and leakage outlet in the middle of the main channel) changes accordingly. Due to the leakage, the velocity after the crack decreased. As can be seen in Figure 4b, more mass flow was leaked at a higher velocity through a wider crack at the same inlet pressure and pressure drop. Pressure drop rate and mass leakage rate relation for three crack widths (50, 100, and 200 μm) were summarized in Figure 5a. With the increase of $\Delta P/P_{in}$, both $\delta \dot{m}$ and ${\dot{m}}_{1}$ increase. However, the mass leakage rate, $\delta \dot{m}/{\dot{m}}_{1}$ decreases linearly with the increase of $\Delta P/P_{in}$. Besides, we conducted a comparison study between incompressible and compressible (ideal gas) model in Figure 5b. As can be seen, the compressibility of gas slightly reduces the mass leakage rate in a high inlet pressure (*P_in_* = 10 kPa) and low pressure drop rate ($\Delta P/P_{in}=5\%$) flow case. The influence is caused by the high velocity in the leakage channel. In this case, although the velocity in the main channel is low (Ma ~ 0.01), the velocity in the leakage channel can reach up to Ma ~ 0.3. Further research on the high Mach number and compressibility influence can be seen in [13].

## 5. Evaluation of Leakage

In this part, based on the virtual entropy generation (VEG) analysis above, we introduced a VEG leakage coefficient Ω_v_ (Ω_v_ = *β*/*α*). Although this coefficient was derived from small leakage assumption in Equation (9), it provides a direction of a large leakage (with a large Ω_v_). In the following study, we also used it in the evaluation of large leakage. In our understanding, the pressure drop rate (*α*) stands for the fluid transportation cost; the mass leakage rate (*β*) describes the economic losses in transportation. Their ratio, VEG leakage coefficient Ω_v_, describes the leakage loss at unit fluid transportation cost.

### 5.1. Case in Biology

(a) Capillary exchange

This case is an evaluation of capillaries exchange with surrounding interstitial fluid in biology [14]. The capillary exists between the arterial end (inlet) and venous end (outlet). The capillaries in human bodies have permeable walls that allow the exchange of nutrients with the surrounding tissues. Thus, capillary leakage plays a positive and crucial role in maintaining the balance of water, oxygen, ions, and other much-needed nutrients in the human body.

The fluid exchange for a normal person maintained at a level of $\delta \dot{V}\approx 20\;L/\mathrm{day}$. The flow rate of blood pumped out from the heart is about 90 mL/s, assuming all blood goes through capillary for mass exchange. The capillary mass exchange rate can be estimated by $\delta \dot{V}/\dot{V}\approx 0.2\%$. The pressure within the arterial end pressure is approximately 35 mmHg. The ambient tissue pressure, $P_{a}\approx 25\;\mathrm{mmHg}$, which is relative constant, $P_{L}-P_{a}\approx 10\;\mathrm{mmHg}$, the pressure drop along the capillary is ΔP≈10 mmHg. The non-dimensional form of pressure drop is quite large at this microscale leakage, $\Delta P/P_{L}\approx 1$. Thus, the VEG leakage coefficient is Ω_v_ = $(\delta \dot{V}/\dot{V})/\left(\Delta P/P_{L}\right)\approx 0.2\%$. For patients with capillary leakage disease syndrome, the mass leakage rate may be bigger.

### 5.2. Cases in Engineering and Industry

**Figure 6:**
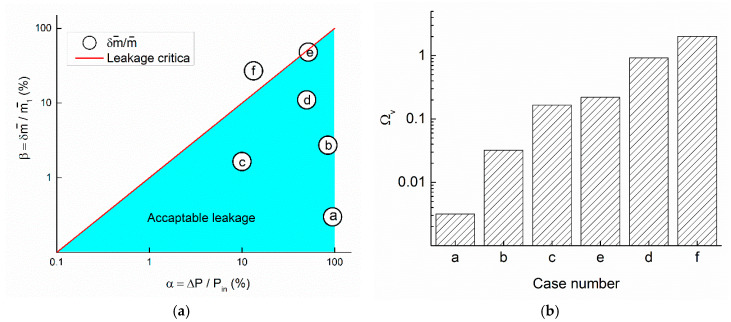
(**a**) Comparison with existing flow leakage process. Leakage of natural gas is about *x* = 10%, y = 3%. Data in are ⓐ capillary exchange; ⓑ smallest detectable leakage; ⓒ a natural gas leakage; ⓓ air preheater; ⓔ hydrogen explosion; and ⓕ crude oil leak incident. (**b**) Leakage vvaluation by VEG leakage coefficient. The *x*-axis is the case number.

Leakage is a significant source of energy waste in industry. Evaluating the economic efficiency may save a significant amount of money. Pipe leakage detection systems typically employ pressure and flow rate measurements to infer the presence of a leak. Available cases are listed and summarized in Figure 6a. Detailed background and calculation of five VEG leakage coefficients are elaborated in the following paragraphs.

(b) Smallest detectable leakage

Leak detection by pressure wave has a limit of detectable leakage due to the attenuation of the pressure wave. In the calculation of smallest detectable leakage [15], the inlet boundary condition is *P_in_* = 2.6 MPa, inlet mass flow rate is ${\dot{m}}_{1}=$ 600 t/h, the smallest detectable leakage amount is about $\delta \dot{m}\approx$ 15 t/day, the mass leakage rate is approximated as $\beta =\delta m/{\dot{m}}_{1}\approx$ 2.73%. The average velocity of the fluid can be estimated from the continuity equation $V=\dot{m/}\rho A$. *A* is the cross-section area of the pipe calculated by $A=\pi R^{2}$, R is the diameter of pipe, *R* = 0.529 m; ρ is the density of the fluid $\rho =847\;\mathrm{kg}/m^{3}$. We estimate the friction coefficient *f* = 0.017 using Moody’s diagram. For a transportation length of 32 km, *P*_out_ ≈ 0.35 MPa. So, the pressure coefficient $\alpha =\Delta P/P_{in}\approx$ 86.5%. The VEG leakage coefficient is Ω_v_ ≈ 0.032.

(c) Leakage in the natural gas transportation

Millions of dollars are lost due to leakage in natural gas transportation. The leakage rate in natural gas transportation is around *β* ≈ 1.65% according to the 2011 Environmental Protection Agency (EPA) report [16]. The leakage coefficient, *α* = Δ*P*/*P_in_* can be considered as the natural gas delivery cost. Case investigation shows a pressure drop for a 100-mile transportation is around 150 psi at 1440 psi inlet pressure [17]. So *α* ≈ 10%, the VEG leakage coefficient can be estimated as Ω_v_ ≈ 0.16.

(d) Leakage in an air preheater

Excessive pressure drop in air preheater can cause a plant shutdown. In an air preheater [18], the leakage from the high-pressure air side went to the low-pressure fuel side. The mass leakage rate is usually within the range of 5% to 15%. In the case of an air preheater leakage: ${\dot{m}}_{1}=136.25\;\mathrm{kg}/s$, δm=15.14 kg/s, $\delta \dot{m}/{\dot{m}}_{1}\approx 11\%$. $P_{in}\approx 2000\;\mathrm{Pa}$. The pressure drop is approximated 1000 Pa for a common pre-heater. So $\Delta P/P_{in}\approx 50\%$. The VEG leakage coefficient is around Ω_v_ ≈ 0.22.

(e) Hydrogen leakage in explosion incident

This is a case of hydrogen gas explosion caused by a pipe leakage [19]. In this case, the hydrogen leakage amount in the explosion was reported up to be 30 kg in the first 30 s. The flow rate of the transportation process is approximate 2600 m^3^/s. The density of hydrogen at 20 bar is *ρ* = 20 × 8.93 mg/L = 1.786 kg/m^3^. The mass leakage rate is about $\delta \dot{m}/{\dot{m}}_{1}\approx 50\%$. In a gas leakage case with $\delta \dot{m}/{\dot{m}}_{1}$ close to 1, the leakage induced pressure change is much bigger than the friction induced pressure drop within a short length of the crack. The leakage induced pressure drop can be estimated by the Clausius–Clapeyron relation through $Pv\propto \dot{m}$. Since nearly half of the gas was leaked, the flow pressure after leakage decreased to one half in a short distance after the crack. The VEG leakage coefficient can be approximated as Ω_v_ ≈ 1.

(f) Crude oil leakage incident

In the Nigerian Bonny Light Crude Oil leak accident [20], *P*_in_ = 120 kPa, the outlet pressure *P*_out_ = 104 kPa, the pressure drop coefficient, $\alpha =\Delta P/P_{in}\approx 13\%$. The inlet mass flow rate is 82.17 kg/s, outlet mass flow rate is 60 kg/s. The leakage rate is 22.17 kg/s, with $\beta =\delta \dot{m}/{\dot{m}}_{1}\approx 27\%$. So, the VEG leakage coefficient is Ω_v_ ≈ 2.

All the data above were summarized in Figure 6a. These data were ranked from (a) to (f) in Figure 6b in a semi-logarithmic coordinate. The oil leakage incident has a value of $\delta \dot{m}/{\dot{m}}_{1}>\Delta P/P_{in}$. From the VEG perspective, the leakage is very serious, non-physical, and unacceptable. For other non-incident leakages, the ratio should be smaller than unity. If this coefficient is close to one, that means the leakage is serious. Otherwise, if it is close to zero, like the capillary exchange, the leakage is negligible. Most industry leakages are much higher than capillary exchange in biology.

## 6. Conclusions

In this paper, we proposed a virtual entropy generation (VEG) criterion for a multiscale leakage evaluation. We showed how the “experimental second-law of thermodynamics” restricts the leakage in a single pipe through mathematical, numerical, and real-life leakage cases. We believe that the virtual entropy generation (VEG) leakage coefficient can be widely used in fluid flow leakage evaluation. Also, VEG methods might help in finding new evaluation parameters in other forms of leakages such as heat flow, electric current, or information leakages.

## Unit Conversions

Leakage rate	1 cfm ≈ 0.4719474 dm^3^/s
Pressure	1 psi ≈ 6894.76 Pa, 1 mmHg ≈ 133.3 Pa, 1 bar = 10^5^ Pa
Length	1 feet ≈ 0.3048 m, 1 mile ≈ 1.609 km
